# Valuable Medical Prescriptions

**Published:** 1881-02

**Authors:** 


Valuable Medical Prescriptions. Frank Harrison & Co., New York. Price 10 cents.

A pamphlet designed for the laity, and rather on the order of such books as "Every One His Own Physician."

